# Kinetic Characterization of an Intestinal Trefoil Factor Receptor

**DOI:** 10.1371/journal.pone.0074669

**Published:** 2013-09-23

**Authors:** Zhang Yong, Wang Lin, Sun Yong, Liang Guang-ping, Wu Dan, Lv Shang-jun, Wu Wei, Peng Xi

**Affiliations:** State Key Laboratory of Trauma, Burns and Combined Injury, Institute of Burns of PLA, Southwest Hospital, Third Military Medical University, Chongqing, PR China; University of Kansas School of Medicine, United States of America

## Abstract

**Objective:**

To determine whether intestinal epithelial cells have a receptor for intestinal trefoil factor and characterize receptor-ligand binding kinetics.

**Methods:**

Radioligand binding assays were performed to characterize the binding kinetics between [^125^I]-labeled ITF and IEC-6, HT-29, Caco2 and HaCaT cells. The *K*_d_, *B_max_* and other kinetic variables describing the interaction between ITF and its potential receptors were determined.

**Results:**

Radioligand binding assays performed at 4°C showed that the *K*_d_ value for the association between [^125^I]-ITF and IEC-6, HT-29, and Caco2 cells were 1.99±0.12×10^−9^ M, 3.89±0.42×10^−9^ M, and 2.04±0.17×10^−9^ M, respectively. *B_max_* values were 1.17±0.04×10^11^, 3.97±0.29×10^11^, and 2.03±0.08×10^11^ sites/cell, respectively. The *K*_i_ values for the interaction between IEC-6, HT-29, and Caco2 cells and non-labeled ITF were 20.98±0.57 nM, 36.87±3.35 nM, and 21.38±0.93 nM, respectively, and the IC_50_ values were 25.21±0.39 nM, 40.68±0.27 nM, and 23.61±0.25 nM, respectively. Radioligand binding kinetic results showed the association rate constants (*k*_+1_) for IEC-6, HT-29, and Caco2 cells were 0.22±0.04 min^−1^, 0.29±0.04 min^−1^, and 0.26±0.05 min^−1^, respectively, and the dissociation rate constants (*k*_-1_) were 0.06±0.02 min^−1^, 0.03±0.01 min^−1^, and 0.04±0.01 min^−1^, respectively. For the HaCaT cells, the *K*_d_ was 4.86±0.28×10^−8^ M and *B*_max_ was 5.81±0.15×10^8^ sites/cell, the very low specific binding between [^125^I]-ITF and these cells made it impossible to calculate binding kinetic parameters.

**Conclusions:**

An ITF-specific receptor appears to be present on the three types of intestinal epithelial cells (IEC-6, HT-29, and Caco-2), and there may be no ITF receptor on epidermal cells.

## Introduction

Intestinal trefoil factor (ITF) is a small peptide secreted by intestinal goblet cells that forms a stable gel complex via the association between specific sites in its spatial structure and polysaccharides in mucin to stabilize the intestinal mucus layer [1]–[3]. ITF can reduce adhesion between cells, accelerate cell migration, and promote mucosal repair by suppressing the expression of cell adhesion molecules [4], [5]. Therefore, ITF plays an important role in intestinal self-defense by mitigating gastrointestinal damage that may be caused by a variety of factors [6], [7]. Although much is known about ITF, few studies have focused on ITF receptors and there remains controversy regarding the existence of ITF-specific receptors.

It has been hypothesized that ITF has no specific receptor. Upon stimulation by ITF, EGFR (Epidermal Growth Factor Receptor) is activated and receptor tyrosine protein kinase activity is enhanced; this led to the suggestion that ITF transmits extracellular signals through EGFR to promote cell proliferation and migration [8]. Other data indicates that ITF can bind to specific cell surface proteins and exert biological effects independently of the EGFR pathway [9], [10]. Chinery et al. found that proteins on MCF-7 and HT-29 cells are capable of binding to ITF, including a 45-kDa non-reducing protein and a 28-kDa reducing protein [11]; however, he did not characterize the proteins further. Using biotin-labeled ITF and ligand blotting, Tan et al. identified a 50-kDa ITF-binding glycoprotein in gastric mucosal cells and intestinal crypt cells [12]. Podolsky claimed the isolation of a specific ITF receptor on the plasma membrane of IEC-6 cells in a U.S. patent application, but showed no evidence that this protein is an ITFR [13]. Thim et al. isolated a 220-kDa ITF binding protein and three 140-kDa ITF-binding proteins from swine gastrointestinal mucosa extracts [14]. Mass spectrometry was used to identify the proteins as CRP-ductin and fibronectin receptor β subunit, but the researchers did not conduct further studies to verify whether the proteins were ITF receptors. Kalabis et al. reported that Vangl plays an important role in the regulation of cell polarity development as an ITF downstream substrate [15], but there is no conclusive evidence to prove Vangl 1 that it is an ITF receptor.

The above studies demonstrated that some proteins can associate with ITF, and some are certainly ITF binding proteins. There is no direct evidence indicate that any of the identified proteins are ITF receptors, however. Here we used radioligand binding assays, a classic receptor research tool, to characterize the interaction between [^125^I]-ITF and a number of cell types. Using the obtained data on receptor affinity, density, and binding kinetics, we sought to determine whether an ITFR is present on intestinal epithelial cells. This study will pave the way for ITFR isolation and characterization of its physicochemical properties.

## Materials and Methods

### Materials

IEC-6 rat intestinal epithelial cells (ATCC Catalog No. CRL-1592), HT-29 and Caco2 human colon cancer cells (ATCC Catalog Nos. HTB-38 and HTB-37, respectively), and HaCaT human epidermal cells (ATCC Catalog No. CRL-2404) were provided by the Institute of Biochemistry and Cell Biology of the Shanghai Biological Sciences, Chinese Academy of Sciences. ITF was expressed and purified (purity above 95%) in our laboratory as previously described [16]. [^125^I]-ITF was labeled by the Institute of Isotope under the China Institute of Atomic Energy to obtain a specific activity of 37.2µCi/µg, a total activity of 120 µCi, and a radiochemical purity of 97.1%. DMEM culture medium and fetal bovine serum (FBS) were purchased from Gibco and trypsin was obtained from Amresco. The FT-608 [^125^I ] radiometer was manufactured by Beijing Medical Instrument Factory.

### Cell culture

IEC-6, HT-29, Caco2, and HaCaT cells were cultured in DMEM medium containing 10% FBS at 37°C in a 5% CO_2_ atmosphere. The culture medium was replaced every other day, and the cells were passaged every 3 to 4 days at a 1:3 ratio. Cells in the sixth to eighth passages were used for experiments.

### Saturation binding experiments

IEC-6, HT-29, Caco2, and HaCaT cells were washed three times with PBS, digested with 0.25% trypsin, mixed thoroughly with serum-free DMEM medium, and adjusted to a concentration of approximately 1×10^6^ cells/mL. The cells were divided into the total binding (TB) group and the non-specific binding (NSB) group; the specific binding (SB) was calculated as the difference between total binding and non-specific binding (SB  =  TB-NSB). For the TB group, [^125^I]-ITF was added to samples each of the four types of cells (2.5×10^5^ cells/tube) to final concentrations of 0.1, 0.2, 0.4, 0.8, 1.6, 3.2, 6.4, and 12.8 pmol/mL. In addition to [^125^I]-ITF, 245 µg unlabeled ITF were added to cells of the NSB group. Serum-free culture medium was added to bring the volume to 0.5 mL, and cells were incubated in a shaker at 4°C for 30 min. Immediately following the incubation, the cells were harvested with suction filtration using glass fiber filters and washed three times with pre-chilled PBS to separate bound and free [^125^I]-ITF. The filters were then loaded into test tubes and radioactivity determined.

### Inhibition experiment

IEC-6, HT-29 and Caco2 cells were washed three times with PBS, digested with 0.25% trypsin, mixed thoroughly with serum-free DMEM medium, and adjusted to a concentration of about 5×10^5^ cells/mL. [^125^I]-ITF was added at a final concentration of 0.4 pmol/mL, and unlabeled ITF was added at final concentrations of 0, 4, 40, 200, 400, 2000 and 4000 pmol/mL. Serum-free media was added to bring the final volume to 0.5 mL, and the samples were incubated in a shaker at 4°C for 30 min. Immediately following the incubation, the cells were harvested with suction filtration using glass fiber filters and washed three times with pre-chilled PBS to separate bound and free [^125^I]-ITF. The filters were loaded into test tubes and radioactivity determined.

### Receptor kinetic experiments

#### Association experiment

IEC-6, HT-29 and Caco2 cells were washed three times with PBS, digested with 0.25% trypsin, mixed thoroughly with serum-free DMEM medium, and counted the cells. 245 µg unlabeled ITF and one of the above three types of cells (4×10^5^ cells/tube) were added. Afterwards, [^125^I]-ITF was added at a final concentration of 0.4 pmol/mL at the time of 2, 3, 4, 6, 8, 10, 13, 16 and 20 min in a shaker at 4°C. Serum-free media was added to bring the final volume to 0.5 mL. Immediately following the incubation, the cells were harvested with suction filtration using glass fiber filters and washed three times with pre-chilled PBS to separate bound and free [^125^I]-ITF. The filters were loaded into test tubes and radioactivity determined.

#### Dissociation experiment

Cultured IEC-6, HT-29 and Caco2 were washed three times with PBS, digested with 0.25% trypsin, mixed thoroughly with serum-free DMEM medium and adjusted to a concentration of about 5×10^5^ cells/mL. [^125^I]-ITF was added at a final concentration of 0.4 pmol/mL, and unlabeled ITF was added at final concentration of 0,4, 40, 200, 400, 2000 and 4000 pmol/mL. Serum-free media was added to bring the final volume to 0.5 mL, and the samples were incubated in a shaker at 4°C for 30 min. Immediately following the incubation, the cells were harvested with suction filtration using glass fiber filters and washed three times with pre-chilled PBS to separate bound and free [^125^I]-ITF. The filters were loaded into test tubes and radioactivity determined.

### Statistical analyses

Analyses were performed using Prism (Version 5.01, GraphPad Software Inc. San Diego, CA USA). Nonlinear regression analysis (assuming one site, specific binding with Hill slope) was conducted for saturation experimental data to determine *K_d_* and *B_max_*. Inhibition test results were analyzed with non-linear regression analysis (assuming one site binding fit to logIC_50_) to obtain *K_i_* and IC_50_. Binding and dissociation equilibrium constants, *K_+1_* and *K_−1_*, respectively were determined by nonlinear regression analysis using the equation for exponential association, and by nonlinear regression analysis. Data were presented as mean ± SEM. Data were analyzed by analysis of variance (ANOVA). All statistical analyses were done using the statistical software program SPSS (Version 18.0), with *p*<0.05 considered significant.

## Results

### Saturation binding experiments

The total binding (TB) of [^125^I]-ITF to IEC-6, HT-29, and Caco2 cells increased with increasing concentrations of [^125^I]-ITF, whereas non-specific binding (NSB) was observed at low levels and was not dependent on the concentration of [^125^I]-ITF. At concentrations higher than 6.4 pmol/mL specific binding (SB) began to be saturated and leveled off when the [^125^I]-ITF concentration was higher than 12.8 pmol/mL (Figures 1.1–1.3). These data suggest that there is a specific receptor for ITF on intestinal epithelial cells. The total binding (TB) of [^125^I]-ITF to HaCaT cells also rose dramatically with increasing concentration of [^125^I]-ITF, but the non-specific binding curve almost overlapped the specific binding curve indicating that there was little specific binding of ITF to epidermal cells (Figure 1.4). The binding affinities (*K_d_*) and maximum number of binding sites (*B_max_*) for ITF on the four types of cells are presented in Table 1.

**Figure 1:**
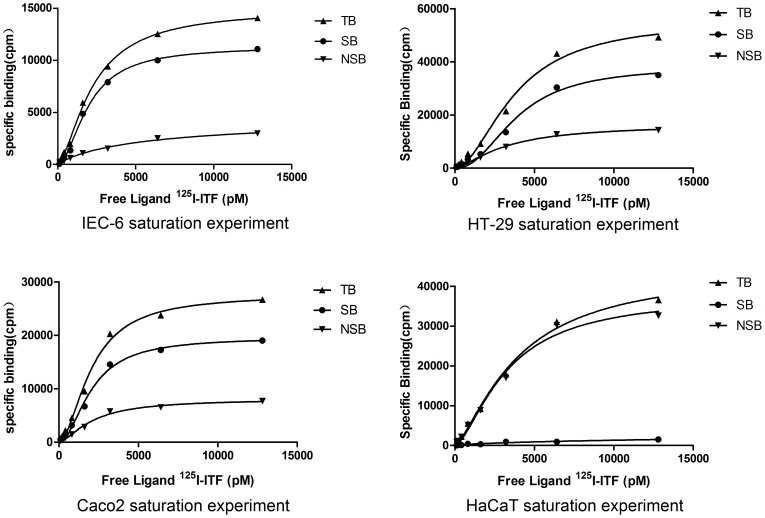
Analyses of binding of ITF to intestinal epithelial cells and to epidermal cells.

**Table 1:** Receptor-ligand binding affinities and maximum numbers of binding sites for ITF on epithelial and epidermal cells(n = 6).

Cell Line	K_d(M)_	B_max(sites/cell)_
IEC-6	1.99±0.12×10^−9^ ** ^##^	1.17±0.04×10^11^ ** ^##^
HT-29	3.89±0.42×10^−9^ **	3.97±0.29×10^11^ **
Caco2	2.04±0.17×10^−9^ ** ^##^	2.03±0.08×10^11^ ** ^##^
HaCaT	4.86±0.28×10^−8 ##^	5.81±0.15×10^8 ##^

** *P*<0.01 compared with HaCaT cells; ***^##^*** *P*<0.01 compared with HT-29 cells;

### Inhibition experiments

The binding between [^125^I]-ITF and epithelial cells gradually decreased and leveled off when the concentration of unlabeled ITF reached 5,000 to 10,000 times (2,000 to 4,000 pmol/mL) the concentration of [^125^I]-ITF (Figure 2.1–2.3). The values for *K_i_* and IC_50_ for the binding of [^125^I]-ITF to IEC-6, HT-29, and Caco2 cells are shown in Table 2.

**Figure 2:**
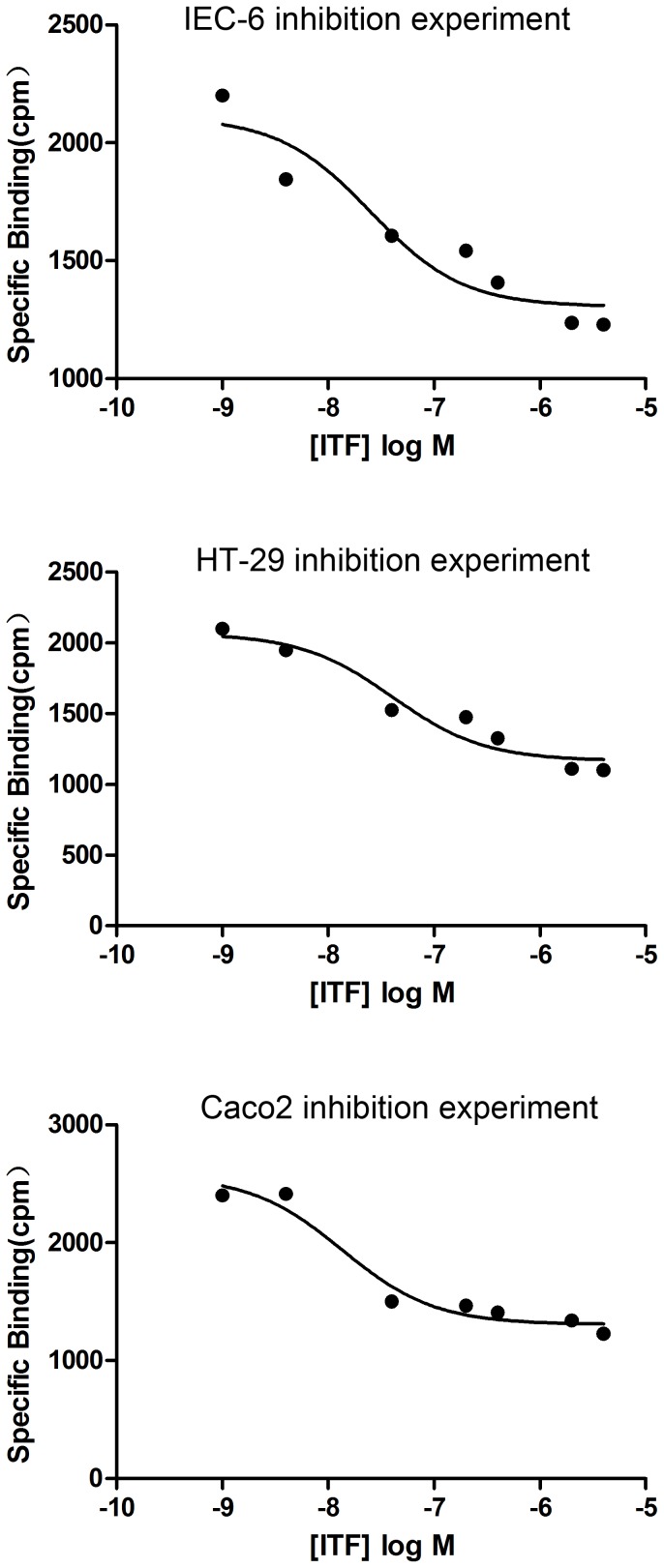
Competition experiment with unlabeled ligand with epithelial cells.

**Table 2:** Receptor-ligand binding affinities and IC_50_ values for epithelial cells (n = 6).

Cell Line	IC_50_ (nM)	K_i_ (nM)
IEC-6	25.21±0.39##	20.98±0.57 ##
HT-29	40.68±0.27	36.87±3.35
Caco2	23.61±0.25 ##	21.38±0.93 ##

## *P*<0.01 compared with HT-29.

### Radioligand-receptor kinetic parameters

The binding of [^125^I]-ITF with IEC-6, HT-29, and Caco2 cells gradually increased over time, with the most significant change occurring in the first 6 min, as evidenced by the maximum slope of the curve. The reaction was equilibrated at 20 min (Figures 3.1-3.3). In dissociation experiments, the addition of a high concentration of unlabeled ITF to IEC-6, HT-29, and Caco2 cells resulted in a significant decrease in the binding of [^125^I]-ITF; the time required for dissociation of half of the [^125^I]-ITF was 17 min, 19 min, and 8.7 min, respectively (Figure 4.1–4.3). The association rate constants (*K*_+1_) and dissociation rate constants (*K_−1_*) for ITF with the epithelial cells are listed in Table 3.

**Figure 3:**
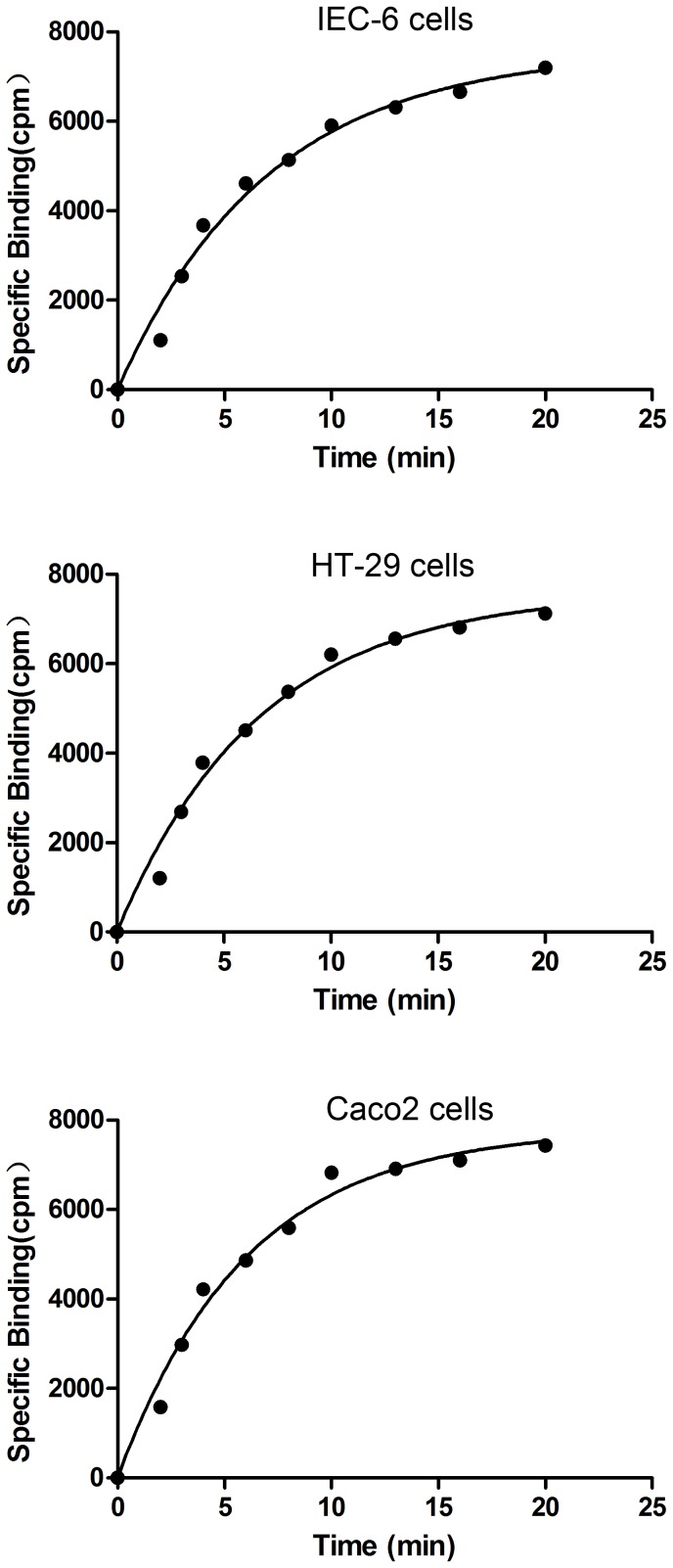
Association of ITF with epithelial cells.

**Figure 4:**
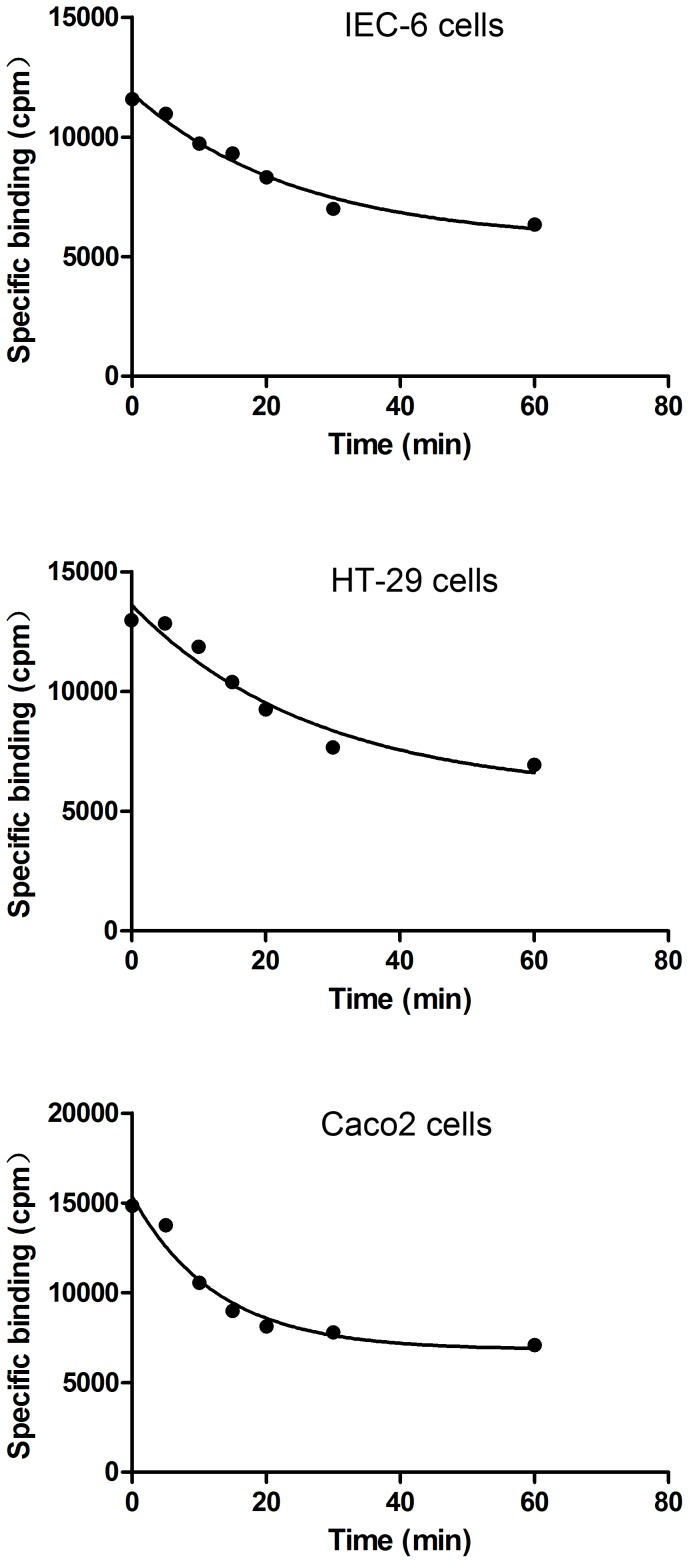
Dissociation of ITF from epithelial cells.

**Table 3:** Association rate constants and dissociation rate constants for ITF binding with epithelial cells (n = 6).

Cell Line	K_+1_ (_min_^−1^)	K _−1_ (_min_^−1^)
IEC-6	0.22±0.04##	0.06±0.02##
HT-29	0.29±0.04	0.03±0.01
Caco2	0.26±0.05	0.04±0.01

## *P*<0.01 compared with HT-29.

## Discussion

The radioligand binding assay can be used to explore receptor characteristics on cells as it can be used to measure formation of a complex of a radioactively labeled ligand with receptors on a cell surface. This technique has been used to determine receptor density, affinity, and dissociation constants. Reaction time, reaction temperature, the number of cells, and relative concentrations of labeled and unlabeled ligands impact results [17], [18]. In general, the number of cells should be more than 10^5^/mL, the time for reaction equilibrium should be 30-60 min, and the relative concentrations of unlabeled to labeled ligand should be over 4000∶1. In this study, we chose a cell concentration of 8×10^5^ cells/mL, a relative concentration of 5000∶1 or 10000∶1 for unlabeled:labeled ligands, and monitored reactions for 30 min at a temperature of 4°C. In pilot experiments at 37°C maximal binding occurred in 10 min and dropped rapidly at 30 min. Because of rapid binding and dissociation at this temperature, data was less reliable than that collected at 4°C. At 4°C, maximum binding occurred at about 30 min, and binding was stable until 60 min. For this reason, 4°C was chosen as the optimal incubation temperature in this study.

In the present study, binding of [^125^I]-ITF to rat intestinal epithelial cells (IEC-6 cells), to human colon epithelial cells derived from carcinomas (HT-29 and Caco2), and to epidermal cells (HaCaT) were evaluated. The results showed that [^125^I]-ITF bound specifically to IEC-6, HT-29, and Caco2 cells: Binding increased as the concentration of the radio-labeled ligand increased and ultimately reached equilibrium with dissociation equilibrium constants on the order of 10^−9^ M. These findings suggest that ITF has high binding affinity for a protein on the surface of intestinal epithelial cells, which is consistent with the basic pattern of classical receptor ligand binding. In contrast, the affinity between ITF and HaCaT cells was significantly lower with a *K*_d_ of 4.86±0.28×10^−8^ M. This result suggests the binding between ITF and a specific protein on the plasma membrane of intestinal epithelial cells is specific receptor-ligand binding. The affinity for binding between ITF and the epidermal cells does not meet the criteria for receptor-ligand binding and is likely adhesion as a result of non-specific binding.

Non-linear regression analysis was used to calculate the number of binding sites per cell. The values for *B_max_* on IEC-6, HT-29, and Caco2 cells were 1.17±0.04×10^11^ sites/cell, 3.97±0.29×10^11^ sites/cell, and 2.03±0.08×10^11^ sites/cell, respectively. Since the ligand is a recombinant human protein, it was not unexpected that the human intestinal epithelial cells have significantly more ITF-binding sites than the rat intestinal epithelial cells. The *B_max_* for HaCaT cells was 5.81±0.15×10^8^ sites/cell, three orders of magnitude lower than those for intestinal epithelial cells. The intestinal epithelial cells had significantly higher affinity for ITF and contained more binding sites for ITF than did the epidermal cells, suggesting that an ITF receptor may be present on the intestinal epithelial cells but not on the epidermal cells.

The two parameters *Ki* and IC_50_ determined in the inhibition tests also suggest that ITF binds specifically to the intestinal epithelial cells. Unlabeled ITF competitively inhibited the binding between [^125^I]-ITF and the cell membrane when the unlabeled factor was present at 5,000 times the concentration of [^125^I]-ITF. The *K_i_* values for IEC-6, HT-29, and Caco2 cells were in the range of 10^−9^ M, and the IC_50_ values were 25.21±0.39 nM, 40.68±0.27 nM, and 23.61±0.25 nM, respectively. As both these values are in the nmol/L level, this confirms the high affinity between ITF and intestinal epithelial cells. The very low specific binding between [^125^I]-ITF and HaCaT cells made it impossible to calculate binding kinetic parameters between the epidermal cells and ITF. These findings indicate that the binding of ITF with a specific protein on the cells is receptor - ligand binding and that different species and types of intestinal epithelial cells have a different binding capacity with ITF.

Our results show that the binding between ITF and its receptor on epithelial cells reached equilibrium after 30 min. The non-radioactive ligand at large doses competed with the radioactive ligand, leading to significant reduction in bound [^125^I]-ITF. The binding reached a new equilibrium after 20 min, which is consistent with the basic characteristics expected for a receptor-ligand interaction. Of the three types of intestinal epithelial cells evaluated, HT-29 cells had the highest *K_+1_* and lowest *K_−1_*, suggesting that these cells have higher affinity for ITF than the other two types of epithelial cells tested. HT-29 cells required significant less time to reach equilibrium and more time for ITF to dissociate than IEC-6 cells. IEC-6 cells are rat cells and we used a recombinant human ITF, which may explain their lower affinity.

These findings strongly suggest the existence of an ITF-specific binding receptor on the plasma membrane of intestinal epithelial cells. However, the identity of this receptor is unknown. Some studies reported that ITF can transmit extracellular signals and promote cell proliferation and migration through EGFR activation [19]–[21]. As there are a large number of EGFR on HaCaT epidermal cells and the binding between [^125^I]-ITF and HaCaT cells do not fit the pattern of receptor-ligand binding, we believe it unlikely that EGFR is the specific ITF receptor. Our results suggest that an ITF-specific receptor is present on the intestinal epithelial cells and that the binding between this receptor and ITF is specific, saturable, and reversible, which is typical of ligand-receptor binding. Future research is needed to identify this receptor and uncover its other biological properties. Our studies lay a solid foundation for isolation, characterization, and functional analysis of the receptor and open up a new avenue for clarifying the mechanism of ITF-conferred intestinal mucosal protection.
